# Rapid Progression of a Urinary Bladder Leiomyosarcoma: Report of a Case

**DOI:** 10.1155/2011/532081

**Published:** 2011-07-06

**Authors:** Toru Yamada, Shingo Nagai, Yusuke Kanimoto

**Affiliations:** Department of Urology, Kakegawa Municipal General Hospital, Shizuoka-ken 436-8502, Japan

## Abstract

The case we report shows rapid progression and a very poor prognosis only for a month that differs from the clinical course reported in the literature. An 83-year-old man was referred to our hospital for macroscopic hematuria. Computed tomography (CT) revealed a large bladder tumor measuring 4 cm × 3 cm and magnetic resonance imaging revealed extravesical invasion and pelvic wall invasion of the tumors. Chest CT and bone scintigraphy revealed no evidence of distant visceral metastases, and a clinical diagnosis of T4N0M0 was made. Transurethral resection of the bladder tumor (TUR-BT) was performed for histopathological diagnosis 18 days after admission, and no further adjuvant treatment was given. At 15 days after TUR-BT, the patient's clinical status worsened with symptoms of exertional dyspnea. CT showed multiple metastatic lesions in the lung, liver, and retroperitoneal lymphadenopathy. The patient died 2 days later and underwent autopsy. A final histopathological diagnosis of leiomyosarcoma was made based on immunohistochemical staining.

## 1. Introduction

Nonepithelial urinary bladder tumors account for less than 5% of bladder malignancies overall, with leiomyosarcoma comprising 0.1% of all bladder cancers [1]. There is a lack of consensus on a standard treatment, and little is known about the natural history and prognosis of urinary bladder leiomyosarcoma due to its very low incidence. The case we report shows rapid progression and a very poor prognosis only for a month that differs from the clinical course reported in the literature.

## 2. Case report

**Figure 1:**
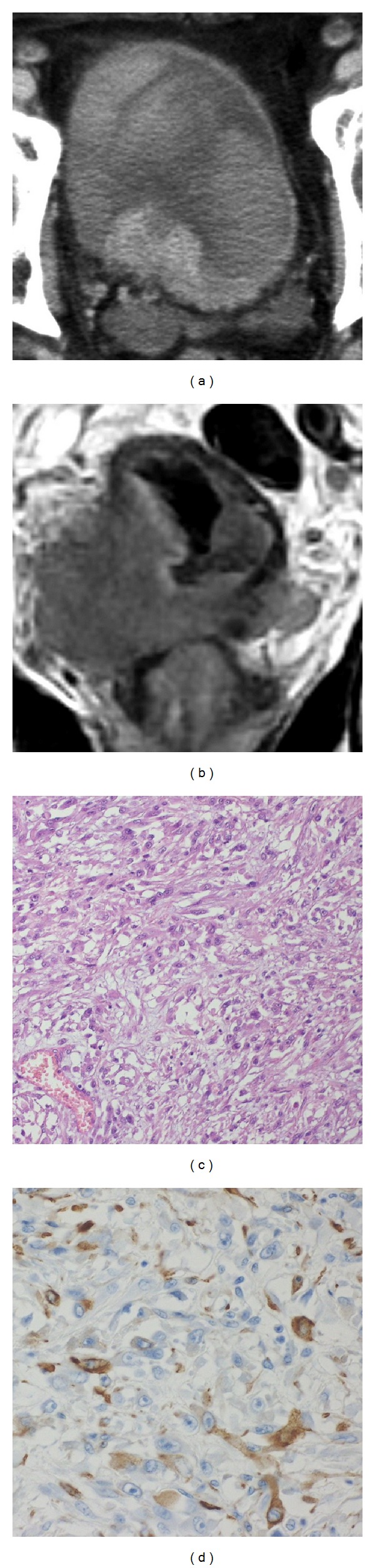
(a) Computed tomography shows a large bladder tumor measuring 4 cm × 3 cm and right hydronephrosis. (b) Magnetic resonance imaging shows extravesical invasion of the tumors. (c) Hematoxylin-eosin stain shows leiomyosarcoma of the bladder tumor. (d) Immunohistochemical staining of the bladder tumor is positive for *α*-smooth muscle actin.

An 83-year-old man was referred to our hospital for macroscopic hematuria and dysuria, and his general condition was poor. Cystoscopy revealed a large broad-based non-papillary bladder tumor on the right bladder wall. Computed tomography (CT) revealed a large bladder tumor measuring 4 cm × 3 cm and right hydronephrosis (Figure 1(a)), and magnetic resonance imaging revealed extravesical invasion and pelvic wall invasion of the tumors (Figure 1(b)). Chest CT and bone scintigraphy revealed no evidence of distant visceral metastases, and a clinical diagnosis of T4N0M0 was made.

Transurethral resection of the bladder tumor (TUR-BT) was performed for histopathological diagnosis 18 days after admission, and Leiomyosarcoma was suspected histopathologically. Because of his advanced age and poor performance status, radical cystectomy was not available and no further adjuvant treatment was given. At 15 days after TUR-BT, the patient's clinical status worsened with symptoms of exertional dyspnea. CT showed multiple metastatic lesions in the lung, liver, and retroperitoneal lymphadenopathy. The patient died 2 days later and underwent autopsy. The autopsy revealed macroscopic multiple metastatic lesions in the bilateral lungs, pleura, diaphragm and liver, and retroperitoneal lymphadenopathy. A final histopathological diagnosis of leiomyosarcoma was made based on immunohistochemical staining, which revealed positive staining for *α*-smooth muscle actin and vimentin and negative staining for cytokeratin (Figures 1(c) and 1(d)).

## 3. Discussion

Urinary bladder leiomyosarcoma is relatively rare, with few large series reported in the literature. Rosser et al. [2] reported 36 cases, and Lee et al. [3] reported 20 cases of urinary bladder leiomyosarcoma. According to Rosser et al. [2], the most common clinical presentation is gross hematuria (81%) followed by dysuria (19%) and pollakiuria (28%). Our patient was referred to us with macroscopic hematuria, but he had no symptoms until the tumor reached an advanced stage and became locally invasive.

Urinary bladder leiomyosarcomas have always been considered highly aggressive tumors that require aggressive surgical extirpation, and radical cystectomy with wide margins should be performed whenever possible [4, 5]. Neoadjuvant and adjuvant therapies were used in 21% and 16% of patients at MD Anderson, respectively, and both resulted in a doubling of disease-specific survival. However, this result was not statistically significant, reflecting the small number of patients in each group. Similarly, it is difficult to evaluate the impact of neoadjuvant and adjuvant chemotherapy on quality of life [4, 5]. Patients with positive surgical margin could be candidates for adjuvant radiotherapy. Patients with local recurrence or metastatic bladder sarcomas should be treated with systemic chemotherapy (as sarcoma chemotherapy protocol using doxorubicin, ifosfamide, cisplatin, and docetaxel), and/or radiotherapy [4, 5]. Contemporary studies suggest that these tumors may have a better prognosis than once believed and show remarkable 5-year-disease-specific survival rates of 59–62% [2, 4].

Some studies suggest that patients with bladder leiomyosarcoma with low mitotic activity (<5 per 10 HPF) and mild-to-moderate nuclear atypia have a good prognosis, whereas those with higher mitotic activity (≧5 per 10 HPF) have a worse prognosis [3]. In our patient, the leiomyosarcoma was diagnosed with high grade mitotic activity (≧10 per 10 HPF). However, no statistically relevant evidence regarding therapeutic behavior can be found in the literature. Therefore, present treatment should be tailored on a case-by-case basis. Our patient showed unexpectedly rapid progression and a very poor prognosis after TUR-BT; therefore, if feasible, aggressive surgical excision and adjuvant therapy should be performed as early as possible in cases of bladder leiomyosarcoma.
